# Comparison of cutting and pencil-point spinal needle in spinal anesthesia regarding postdural puncture headache: A meta-analysis

**DOI:** 10.1097/MD.0000000000006527

**Published:** 2017-04-07

**Authors:** Hong Xu, Yang Liu, WenYe Song, ShunLi Kan, FeiFei Liu, Di Zhang, GuangZhi Ning, ShiQing Feng

**Affiliations:** Department of Orthopaedics, Tianjin Medical University General Hospital, Heping District, Tianjin, People's Republic of China.

**Keywords:** cutting spinal needle, lumbar puncture, meta-analysis, pencil-point spinal needle, postdural puncture headache, spinal anesthesia

## Abstract

**Background:**

Postdural puncture headache (PDPH), mainly resulting from the loss of cerebral spinal fluid (CSF), is a well-known iatrogenic complication of spinal anesthesia and diagnostic lumbar puncture. Spinal needles have been modified to minimize complications. Modifiable risk factors of PDPH mainly included needle size and needle shape. However, whether the incidence of PDPH is significantly different between cutting-point and pencil-point needles was controversial. Then we did a meta-analysis to assess the incidence of PDPH of cutting spinal needle and pencil-point spinal needle.

**Methods:**

We included all randomly designed trials, assessing the clinical outcomes in patients given elective spinal anesthesia or diagnostic lumbar puncture with either cutting or pencil-point spinal needle as eligible studies. All selected studies and the risk of bias of them were assessed by 2 investigators. Clinical outcomes including success rates, frequency of PDPH, reported severe PDPH, and the use of epidural blood patch (EBP) were recorded as primary results. Results were evaluated using risk ratio (RR) with 95% confidence interval (CI) for dichotomous variables. Rev Man software (version 5.3) was used to analyze all appropriate data.

**Results:**

Twenty-five randomized controlled trials (RCTs) were included in our study. The analysis result revealed that pencil-point spinal needle would result in lower rate of PDPH (RR 2.50; 95% CI [1.96, 3.19]; *P* < 0.00001) and severe PDPH (RR 3.27; 95% CI [2.15, 4.96]; *P* < 0.00001). Furthermore, EBP was less used in pencil-point spine needle group (RR 3.69; 95% CI [1.96, 6.95]; *P* < 0.0001).

**Conclusions:**

Current evidences suggest that pencil-point spinal needle was significantly superior compared with cutting spinal needle regarding the frequency of PDPH, PDPH severity, and the use of EBP. In view of this, we recommend the use of pencil-point spinal needle in spinal anesthesia and lumbar puncture.

## 1 Introduction

Spinal anesthesia is one of the commonest techniques used in anesthetic practice in obstetric patients, children, inpatients, and ambulatory surgery patients. Needle design variables, such as the needle size and needle shape, have been modified to enable rapid flow of cerebral spinal fluid (CSF) and injected medications, yet simultaneously limit dural trauma and loss of CSF.^[1]^ A headache occurring within 5 days after lumbar puncture, and being aggravated when standing or sitting and relived when lying flat, is defined as postdural puncture headache (PDPH) on the grounds of the International Classification of Headache Disorder, 3rd edition.^[2]^ PDPH is a well-known iatrogenic complication of spinal anesthesia, which continues to be a major problem.^[3–6]^ It is the drawback to the use of spinal anesthesia or diagnostic lumbar puncture,^[1,7]^ resulted from the loss of CSF and the following tension on meninges aroused by the hole created in the dural tissues.^[1]^ PDPH was usually mild with no limitation of activity and required no treatment while patients with severe PDPH were confined to bed. An epidural blood patch (EBP), injecting the blood of the patients own into the epidural space to patch the hole created in the dural tissues, was often used to treat severe PDPH.^[8]^

Modifiable risk factors of PDPH included the needle size, needle shape, bevel orientation and inserting angle, stylet replacement, and operator experience.^[9]^ Needle size might be the most significant factor in the development of PDPH.^[3,10,11]^ Spinal needles generally used today are 22 to 27 G, but sizes ranging from 19 to 30 G are available.^[1]^ The incidence of PDPH after spinal anesthesia performed with Quincke, an cutting needle, is 36% with 22 G needle, 25% with 25 G needle, 2% to 12% with 26 G needle, and less than 2% for smaller than 26 G needles.^[3,11–14]^ The smaller needle diameter reduces the incidence of PDPH.^[9,15]^ However, even the use of 29 G needles will reduce the complication, they are too thin to use.^[10]^ Spinal needle, which is extremely thin (29 G or smaller), would increase the rate of failure for spinal anesthesia. And multiple dural punctures caused by unsuccessful puncture would increase the rate of PDPH.^[10,16–19]^ And sometimes CSF is too viscous to come through a small needle.^[5]^

As for the tip design, the cutting-point needles were easier to insert through the skin and ligaments, while the pencil-point needles were easier to recognize the dura mater.^[20,21]^ Some studies argued that the incidence of PDPH was not significantly different between cutting-point and pencil-point needles^[22,23]^ while some opposited, arguing noncutting needle lead to lower rate of headache.^[24–26]^ A previous meta-analysis published in 2000 has compared the frequency of PDPH between Quincke (a cutting-point spinal needle) and pencil-point spinal needles which suggested that pencil-point spinal needle will significantly reduce PDPH rate compared with Quincke spinal needles.^[24]^ However, only studies with Quincke spinal needle were included, while other cutting spinal needles were ignored. What is more, the amount of patients included was only 313, and the studies quality, PDPH severity and outcomes credibility were not available. Our previous meta-analysis showed Whitacre spinal needle was better than Quincke spinal needle.^[19]^ However, it was also limited as only 9 RCTs were included, and only 2 kinds of spinal needles, Whitacre spinal needle and Quincke spinal needle, were compared.

The goal of this analysis was to firstly find out all types of needle used for spinal anesthesia and lumbar puncture, and then distinguish whether they are cutting or pencil-point. Then, we compared the frequency of PDPH and the rate of severe PDPH in patients given spinal anesthesia with different tip design, and the use of EBP were compared as well. Meanwhile, only randomly controlled trial were included. This analysis aimed to find out the superior spinal needle in spinal anesthesia and lumbar puncture through the Grades of Recommendation, Assessment, Development and Evaluation (GRADE) system.

## 2 Methods

### 2.1 Search strategy

An extensive electronic search for randomized controlled trials (RCTs) was conducted by 2 independent investigators (HX and YL) via the following 3 databases: PubMed, Embase, and Cochrane Central Register of Controlled Trials. The last search was updated on March 31, 2016. To identify the search terms, searches were performed using Medical Subject Headings (MeSH) combined with following free words: “spinal anesthesia”; “lumbar puncture”; “post dural puncture headache”; “epidural blood patch”; “randomized controlled trial”; and the names of various spinal needles. Sixteen kinds of needles (10 cutting and 6 pencil-point) were involved in this study.

### 2.2 Study inclusion and exclusion criteria

Two independent investigators (HX and YL) reviewed the studies that met the following inclusion criteria: the study was randomly controlled trial, the patients were randomly assigned to different groups to receive spinal anesthesia or lumbar puncture with either cutting or pencil-point spinal needle, frequency of PDPH was recorded. Studies were excluded for any of the following reasons: the study was a case report or review article, the study was not about spinal anesthesia or lumbar puncture, the study was not designed to compare cutting spinal needle with pencil-point spinal needle, extremely thin (29 G or smaller) spinal needles which would increase the incidence rate of PDPH were used in the study, only English published studies were included. Any disagreement was resolved by discussion between the 2 investigators (HX and YL).

### 2.3 Study quality assessment

All selected studies were assessed for the risk of bias by referring to the Cochrane Handbook for Systematic Reviews of Interventions for the following 7 domains: random sequence generation (selection bias), allocation concealment (selection bias), blinding of participants and personnel (performance bias), blinding of outcome assessment (detection bias), incomplete outcome data (attrition bias), selective reporting (reporting bias), and other bias. All risks of bias were evaluated with a grade of low or high, and “no data obtained” was recorded if a risk could not be applied to a study.

### 2.4 Potential effect modifiers and reasons for heterogeneity

Risk ratio (RR) and 95% confidence interval (CI) were implemented. The heterogeneity of included studies was assessed and quantified in term of the *I*^2^ statistic on the level of α = 0.05. The fixed-effect model was used if there was no evidence of heterogeneity where *I*^2^ ≤ 50%, otherwise the random-effect model was used. The result robustness was tested by single elimination of each study one by one and insecure studies were excluded where *I*^2^ > 50%. A funnel plot was used to test the potential publication bias if more than 10 studies were included.

### 2.5 Data extraction strategy

The following data were extracted by 2 investigators (HX and YL): year of publication, number of patients, country of origin, characteristics of patients (age, sex, and operation), spinal needle used for spinal anesthesia or lumbar puncture, clinical outcomes including follow-up, frequency of PDPH, reported severe PDPH, and the use of EBP. Meanwhile, as the selected studies assessed the grade of PDPH severity in different criterion, we distinguished the mild and severe PDPH by “necessary periodic bed rest and analgesics for the intolerable headache” uniformly, and cases of PDPH were regarded as severe ones when an EBP were used. Data extraction was done by reading the full article with interpretation of figures and tables in every study included. Disagreements occurred rarely and were resolved through consensus.

### 2.6 Data synthesis and presentation

Rev Man software (version 5.3) was used to pool all appropriate data. Rate of PDPH, severe PDPH, and EBP used were pooled as dichotomous outcomes.

### 2.7 Ethical statement

As all analyses were grounded on previously published studies, no ethical approval was necessary.

## 3 Results

### 3.1 Review statistics

One hundred thirteen articles were yielded via the initial selected database searches. Meanwhile, 2 RCTs meeting our inclusion criteria were added by manually review. Eighty-eight references remained after duplication had been removed. Inclusion criteria and exclusion were strictly implemented. Fifty-four articles met the primary exclusion criteria underwent title/abstract review. This left 34 articles for full-test review. Then, 9 references were excluded, including 4 non-English studies. This left 25 RCTs included for this comprehensive evaluation. The selection process was showed in the flow diagram.

The identified 25 RCTs included 9 RCTs practiced in obstetric patients. The total number of patients included in our review was 6539, with patients ranging from 56 to 965 in each study (Table 1). In these RCTs, totally 3255 patients were performed spinal anesthesia or lumbar puncture with cutting-point spinal needles and 3284 patients with pencil-point ones. The other characteristics of each included study are presented in Table 1.

**Table 1:**
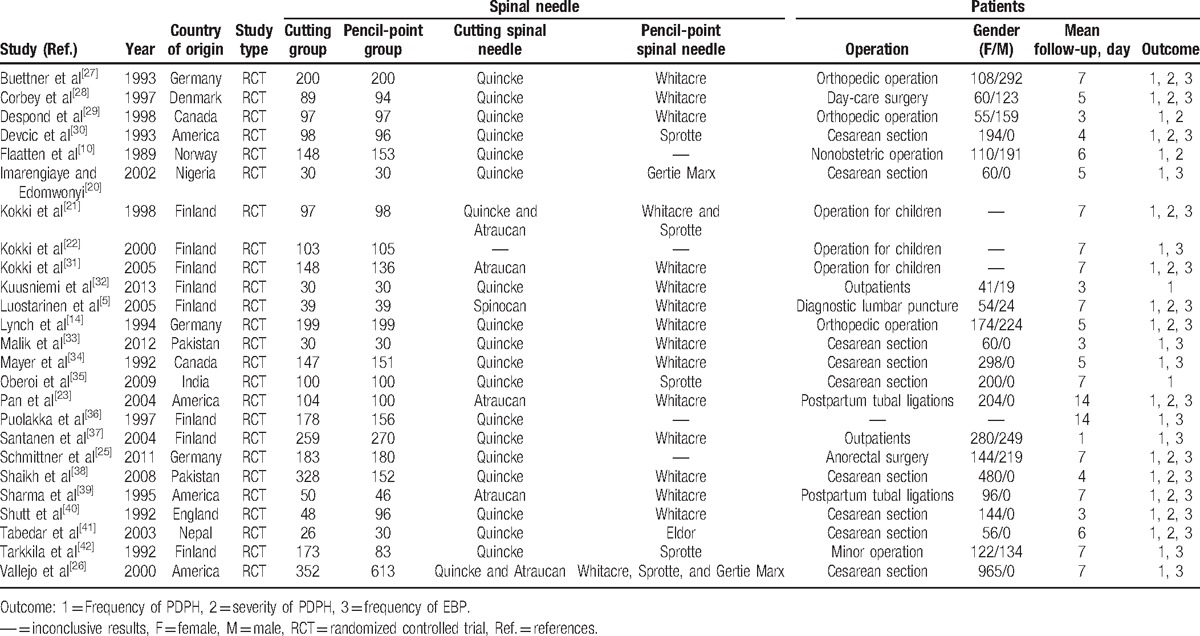
Characteristics of included trials comparing cutting vs. pencil-point spinal needle.

### 3.2 Study quality assessment

**Figure 1:**
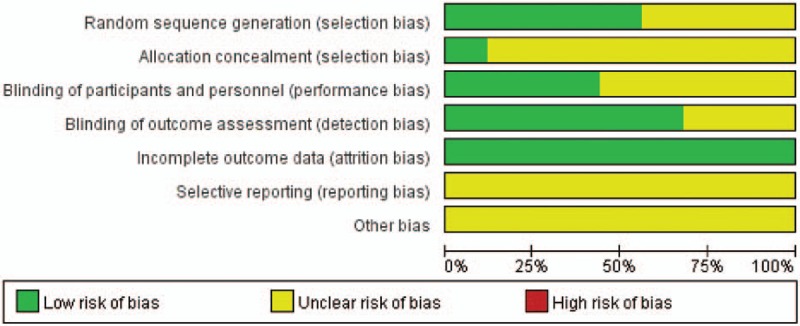
Risk of bias graph for all included RCTs.

**Figure 2:**
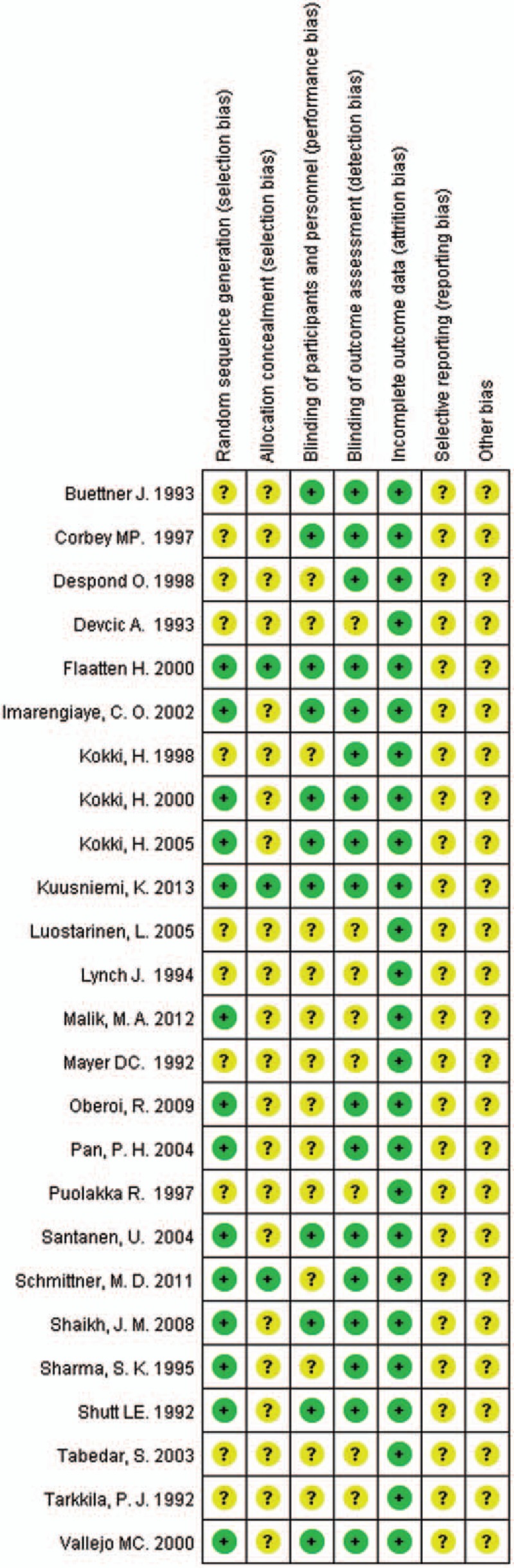
Risk of bias summary for all included RCTs.

Random sequence generation was reported in all RCTs, while 14 of them offered the random method, meaning a low risk of selection bias. However, sealed envelope technique, which was regarded as allocation concealment, was only used in 3 studies. And blinding of participants and personnel, representing low risk of selection bias, was reported to be carried out in eleven RCTs, while blinding of outcome assessment, for detection bias, in 17 RCTs. The loss rate of follow-up was less than 15% in all the included studies, meaning a low risk of attrition bias. And risk of reporting bias was low in all RCTs as well. A review of the authors’ judgment about each risk of bias item is shown in Fig. 1, and the results of each study's risk of bias are listed in Fig. 2.

### 3.3 Frequency of PDPH

**Figure 3:**
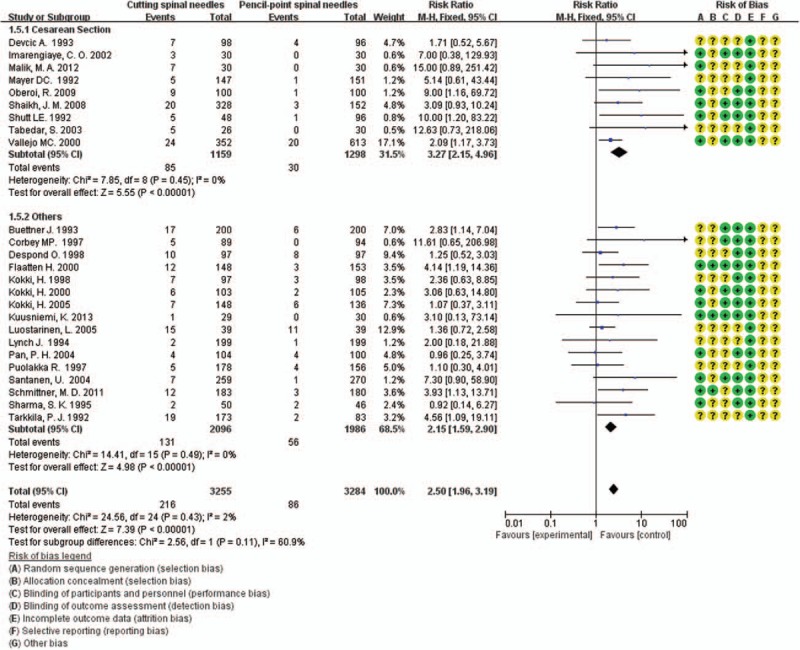
Frequency of PDPH of all pooled RCTs, including 9 performed in obstetric patients.

**Figure 4:**
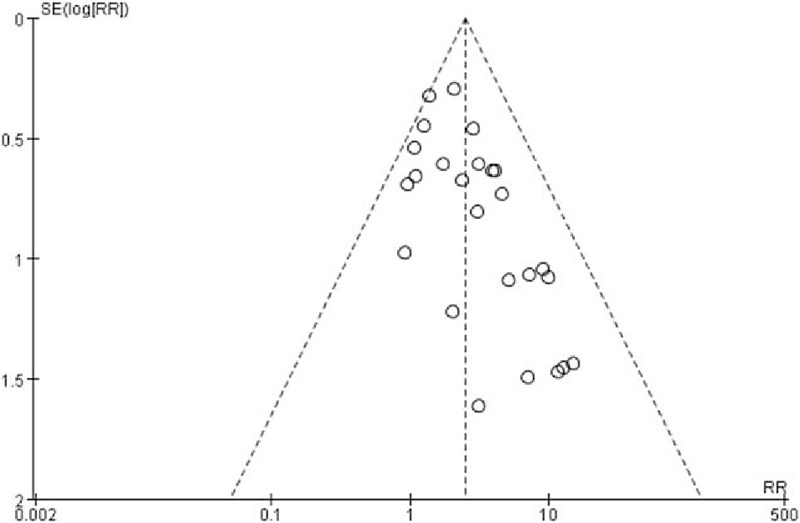
Funnel plot assessing publication bias for all pooled RCTs.

The incidence rates of PDPH after spinal anesthesia or lumbar puncture in cutting-point spinal needle group and pencil-point spine needle group were pooled. In totally 6539 patients, 302 patients (4.6%) were reported having suffered from PDPH.^[5,14,20–42]^ Two hundred sixteen of 3255 patients (6.6%) in cutting-point spinal needle group developed PDPH while 86 of 3284 patients (2.6%) in pencil-point spine needle group developed the same complication. Heterogeneity of the 25 studies were tested and showed no statistical significance (*P* = 0.43; *I*^2^ = 2%). Then the fixed-effect model was performed. The incidence rate of PDPH in the pencil-point spine needle group was significantly lower than that in the cutting-point spinal needle group (RR 2.50; 95% CI [1.96, 3.19]; *P* < 0.00001; Fig. 3). A funnel plot was performed for these 25 RCTs to test the potential publication bias and showed no obvious bias (Fig. 4).

### 3.4 Frequency of PDPH in obstetric patients

PDPH is more frequent in the female gender,^[9,15]^ as women have almost twice the risk of developing a PDPH in comparison with men.^[7,9,43–45]^ It is more common in reproductive age group and pregnant females.^[35]^ Of the 25 RCTs, 9^[20,26,30,33–35,38,40,41]^ were performed in obstetric patients. We made a subgroup to compare the frequency of PDPH in these peculiar patients. These 9 studies enrolled 2457 pregnant women undergoing elective or emergency cesarean delivery under spinal anesthesia, with 1159 patients in cutting spine needle group and 1298 patients in pencil-point spine needle group. The overall rate of PDPH in the entire group was 4.7% (115 of 2457 patients), with 7.3% (85 of 1159 patients) for cutting spine needle group and 2.3% (30 of 1298 patients) for pencil-point spine needle group. The result by the fixed-effect model (*P* = 0.45; *I*^2^ = 0%) showed that the incidence rate of PDPH in the pencil-point spine needle group was significantly lower than that in the cutting spine needle group (RR 3.27; 95% CI [2.15, 4.96]; *P* < 0.00001; Fig. 3).

### 3.5 Severity of PDPH

**Figure 5:**
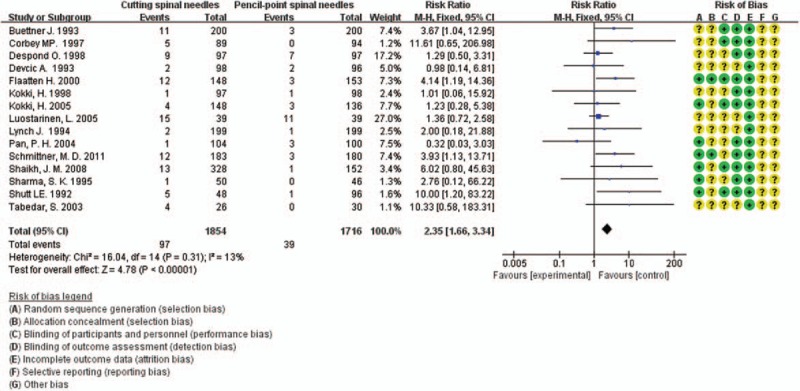
Severity of PDPH of the 15 pooled RCTs.

**Figure 6:**
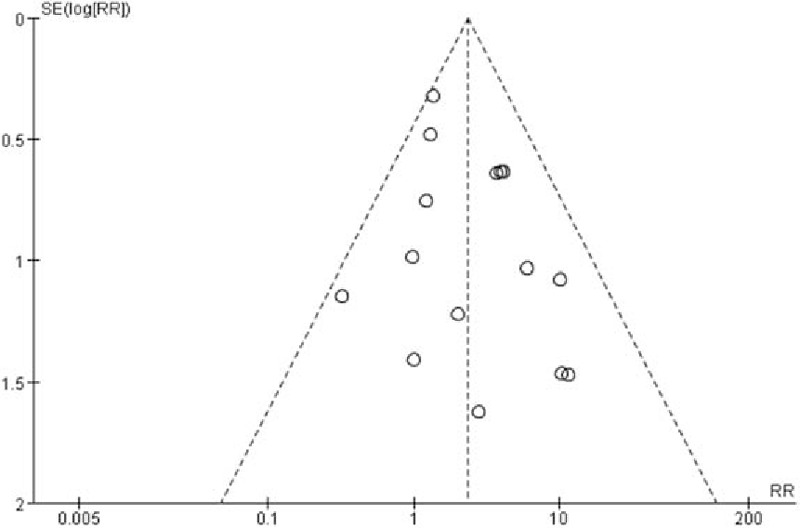
Funnel plot assessing publication bias for the 15 pooled RCTs reporting the severity of PDPH.

Of the 25 RCTs, 15^[5,14,21,23–25,27–31,38–41]^ reported the severity of PDPH which could be graded as mild or severe. These 15 studies enrolled 3570 patients, with 1854 patients in cutting spine needle group and 1716 patients in pencil-point spine needle group. The overall rate of severe case in the entire group was 3.8% (136 of 3570 patients), with 5.2% (97 of 1854 patients) for cutting spine needle group and 2.3% (39 of 1716 patients) for pencil-point spine needle group. The result by the fixed-effect model (*P* = 0.31; *I*^2^ = 13%) showed that the incidence rate of severe PDPH was significantly lower in pencil-point spine needle group than that in the cutting spine needle group (RR 2.35; 95% CI [1.66, 3.34]; *P* < 0.00001; Fig. 5). The funnel plot showed low potential publication bias (Fig. 6).

### 3.6 Frequency of EBP

**Figure 7:**
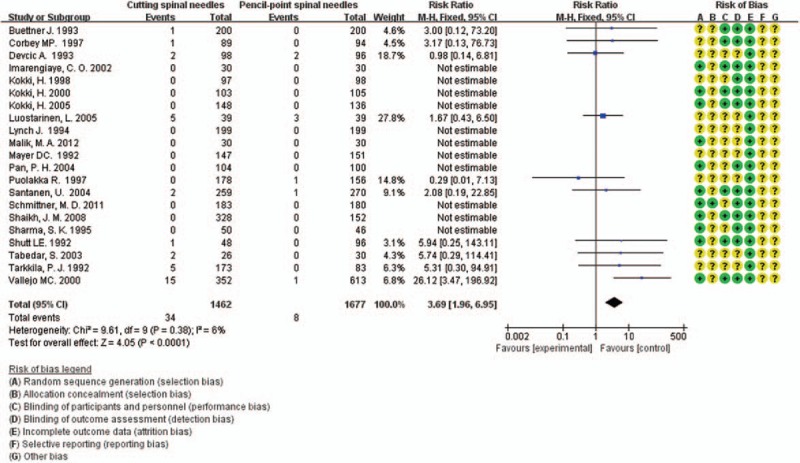
Frequency of the use of EBP of the 10 pooled RCTs.

**Figure 8:**
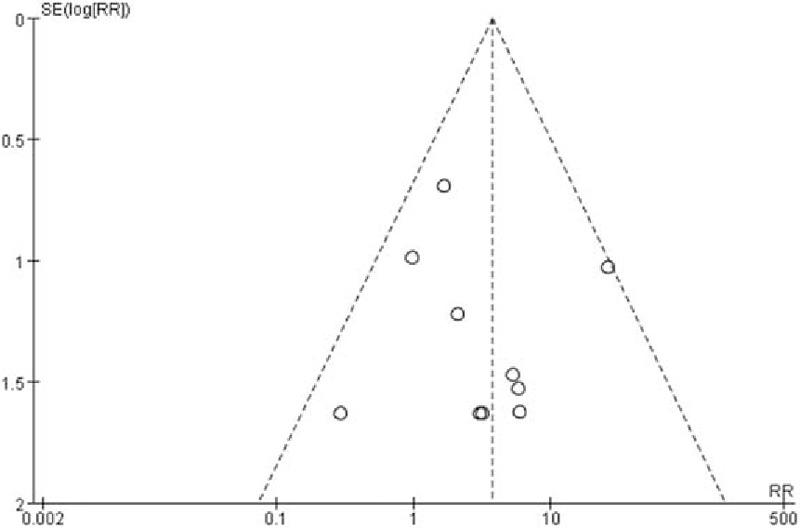
Funnel plot assessing publication bias for the 10 pooled RCTs reporting the use of EBP.

Twenty-three RCTs mentioned the use of EBP while 11 of them reported no use of EBP and 2 did not mention which group they happened in. The remaining 10 studies^[5,26–29,36,37,40–42]^ enrolled 3139 patients, with 1462 patients in cutting spine needle group and 1677 patients in pencil-point spine needle group. The overall rate of EBP in the entire group was 1.3% (42 of 3139 patients), with 2.3% (34 of 1462 patients) for cutting spine needle group and 0.5% (8 of 1677 patients) for pencil-point spine needle group. The result by the fixed-effect model (*P* = 0.38; *I*^2^ = 6%) showed that, in cutting spine needle group, EBP is much more frequently used than in pencil-point spine needle group (RR 3.69; 95% CI [1.96, 6.95]; *P* < 0.0001; Fig. 7). The funnel plot showed low potential publication bias (Fig. 8).

### 3.7 GRADE profile evidence

The GRADE quality of evidence is presented in Table 2.

**Table 2:**
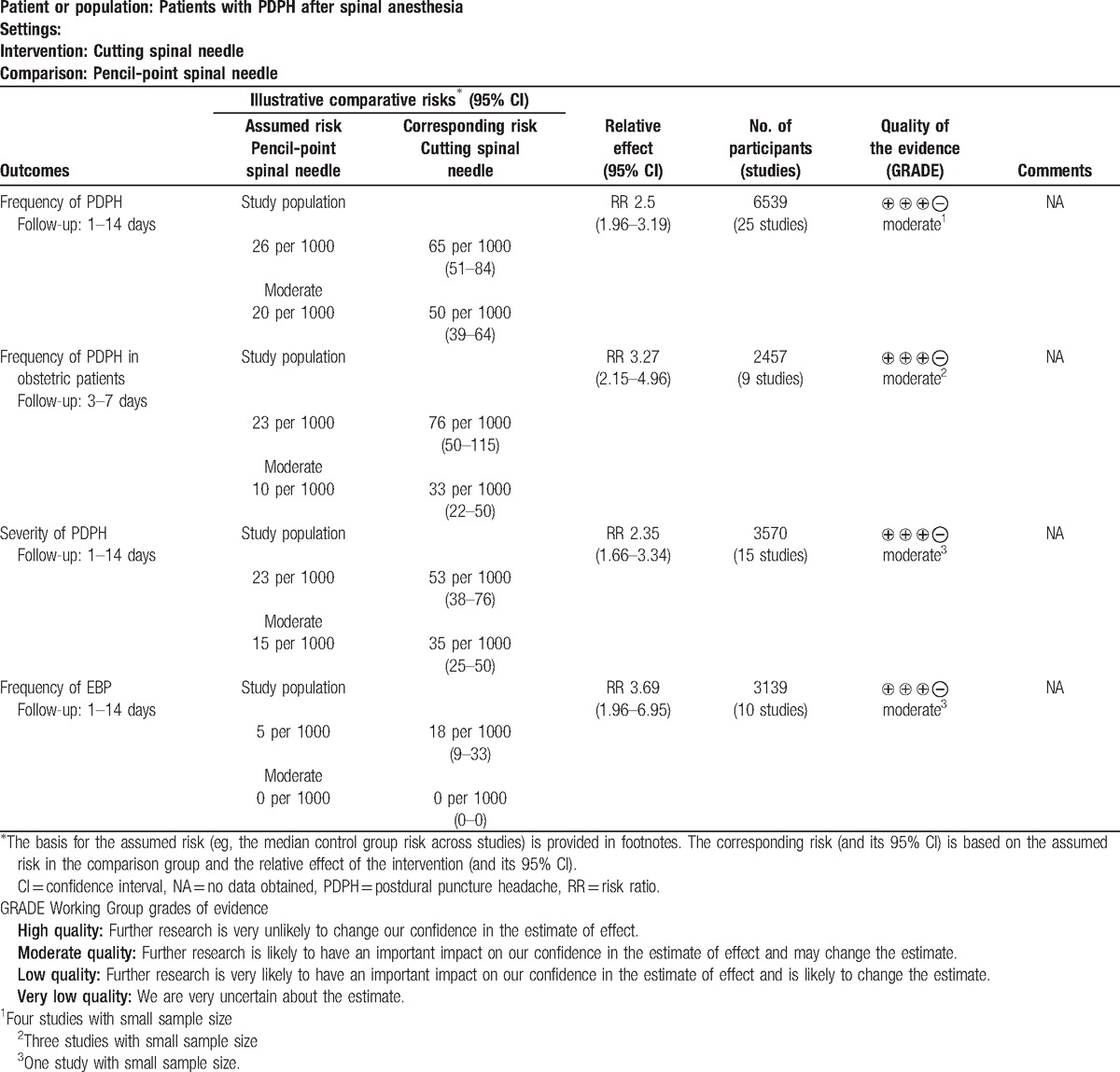
The GRADE evidence profile.

## 4 Discussion

Twenty-five RCTs were included in this meta-analysis. Pencil-point spinal needle was proved to be more appropriate for spinal anesthesia and diagnostic lumbar puncture than cutting spinal needle regarding the frequency of PDPH (2.5 times higher in cutting group). And the difference was more obvious in pregnant females (3.2 times higher in cutting group). Pencil-point spinal needle gave rise to less severe PDPH as well (2.3 times higher in cutting group). Furthermore, while comparing the use of EBP, pencil-point spinal needle was significantly superior (4.6 times higher in cutting group). We further confirmed that pencil-point spinal needle is significantly superior compared with cutting spinal needle. In view of these, we recommend the use of pencil-point spinal needle in spinal anesthesia and lumbar puncture.

We used to compare the performance of Whitacre spinal needle (a kind of pencil-point spinal needle) and Quincke spinal needle (a kind of cutting spinal needle) in the practice of spinal anesthesia or diagnostic lumbar puncture. Our previous meta-analysis showed Whitacre spinal needle was better than Quincke spinal needle.^[19]^ We had further conjectured that pencil-point spinal needle might be better than cutting spinal needle. Therefore we searched all types of spinal needle to compare the effects. And this meta-analysis proved our conjecture.

Needle size is the most significant factor in the development of PDPH.^[3,10,11]^ The smaller needle diameter had been thought effective to reduce the incidence of PDPH.^[9,15]^ However, extremely thin spinal needles would increase the rate of failure for spinal anesthesia, resulting in multiple dural punctures and high incidence rate of PDPH.^[10,16–19]^ Apart from the spinal needle size, spinal needle shape might be the most important modifiable risk factors of PDPH. And we consider pencil-point spinal needle another effective way to reduce the incidence of PDPH in spinal anesthesia and lumbar puncture.

PDPH is resulted from the loss of CSF and the following tension on meninges aroused by the hole created in the dural tissues. We believed that the great performance of pencil-point needle derive from its capability of reducing the damage of dural fibers and the loss of CSF and promoting healing.^[19,35,46]^ This was in line with the guideline of American Academy of Neurology in 2013^[47]^ which recommended the use of pencil-point needles because of lower rates of headache following lumbar puncture in randomized trials.

PDPH is the drawback to the use of spinal anesthesia or diagnostic lumbar puncture.^[1,7]^ It can also be associated with side effects like nausea, vomiting, dizziness, tinnitus, hearing loss, or blurring of vision.^[35,48]^ Patients might chronically suffered from these symptoms for months and even years.^[49]^ Since spinal anesthesia and diagnostic lumbar punctures are indispensable techniques in clinical therapy, minimizing adverse effects and reducing the occurrence of PDPH is of great significance.

Ultimately, this is the first time that all types of spinal needles are compared.

### 4.1 Limitations

Our study was limited in several aspects. First, while collecting data, though having restricted the size of spinal needles from 22 to 27 G, we only depended on the great amount of data to lower the effect of the size. Second, some studies were not quite appropriate because of the small sample size, especially the 4^[32,33,41,47]^ of which with even no more than 30 patients in each group. The robustness of the result was tested by excluding these 4 studies, and no obvious change happened. Third, of all the studies, 14^[5,14,21,22,24,25,27,28,31,32,36,37,40,42]^ were performed in Europe, 6^[23,26,29,30,34,39]^ in North America, 4^[33,35,38,41]^ in Asia, and 1^[20]^ in Africa, reflecting a limitation of the evidence generalization. Lastly, all the documents were in English, meaning the possibility of language bias.

## 5 Conclusion

Current evidences suggest that pencil-point spinal needle is significantly superior comparing with cutting spinal needle regarding the frequency of PDPH, PDPH severity, and the use of EBP. In view of this, we recommend the use of pencil-point spinal needle in spinal anesthesia and lumbar puncture. Studies comparing needles with different tip in same size, studies with larger size of sample, and non-English studies are needed in order to further evaluation.
